# Geophagia and risk of squamous cell esophageal cancer in the African esophageal cancer corridor: Findings from the ESCCAPE multicountry case‐control studies

**DOI:** 10.1002/ijc.33688

**Published:** 2021-05-28

**Authors:** Clement T. Narh, Charles P. Dzamalala, Blandina T. Mmbaga, Diana Menya, Yohannie Mlombe, Peter Finch, Gissela Nyakunga, Joachim Schüz, Valerie McCormack, ESCCAPE team

**Affiliations:** ^1^ Branch of Environment and Lifestyle Epidemiology International Agency for Research on Cancer (IARC) Lyon France; ^2^ School of Public Health University of Health and Allied Sciences Hohoe Ghana; ^3^ College of Medicine University of Malawi Blantyre Malawi; ^4^ Kilimanjaro Clinical Research Institute Moshi Tanzania; ^5^ Kilimanjaro Christian Medical Centre Moshi Tanzania; ^6^ School of Public Heath Moi University Eldoret Kenya

**Keywords:** Africa, cancer epidemiology, geophagia, esophageal squamous cell carcinoma, risk factors

## Abstract

Geophagia, the intentional practice of consuming soil, occurs across the African esophageal cancer corridor, particularly during pregnancy. We investigated whether this practice is linked to endemic esophageal squamous cell carcinoma (ESCC) in this region. We conducted ESCC case‐control studies in Tanzania, Malawi and Kenya. Cases were patients with incident histologically/clinically confirmed ESCC and controls were hospital patients/visitors without digestive diseases. Participants were asked if they had ever eaten soil (never/regularly/pregnancy‐only). Odds ratios (OR) are adjusted for sex, age, tobacco, alcohol, country, religion and marital status. Overall, 934 cases (Malawi 535, Tanzania 304 and Kenya females 95) and 995 controls provided geophagia information. Among controls, ever‐geophagia was common in women (Malawi 49%, Kenya 43% and Tanzania 29%) but not in men (10% Malawi, <1% Tanzania). In women, ESCC ORs were 1.25 (95% CI: 0.70, 2.22) for regular versus never geophagia and 0.88 (95% CI: 0.64, 1.22) for pregnancy‐only versus never. Findings were stronger based on comparisons of cases with hospital visitor controls and were null using hospital patients as controls. In conclusion, geophagia is too rare to contribute to the male ESCC burden in Africa. In women, the practice is common but we did not find consistent evidence of a link to ESCC. The study cannot rule out selection bias masking modest effects. Physical effects of geophagia do not appear to have a large impact on overall ESCC risk. Research with improved constituent‐based geophagia exposure assessment is needed.

**Abbreviations**
AfrECC: African EC consortium
aOR: fully adjusted odds ratio
CI: confidence interval
EC: esophageal cancer
ESCC: esophageal squamous cell carcinoma
ESCCAPE: ESCC African PrEvention research
IARC: International Agency for Research on Cancer
KCMC: Kilimanjaro Christian Medical Centre
mOR: minimally adjusted odds ratio
OR: odds ratio
SD: standard deviation

**What's new?**

The etiological factors giving rise to the African esophageal cancer corridor remain understudied. A hypothesized contributor risk is geophagia, the traditional practice of consuming soil, particularly during pregnancy. In this study, the physical effects of geophagia do not appear to have a large impact on overall esophageal squamous cell carcinoma (ESCC) risk. Regular geophagia was too rare in men, and despite the higher prevalence of this habit in women, there was no consistent evidence of increased ESCC risk. Research in young populations (<50 years) with improved chemical component‐based geophagia exposure assessment is needed.

## 1 INTRODUCTION

Analogous to the Asian esophageal cancer belt, an African esophageal cancer corridor stretches from Ethiopia down to the Eastern Cape of Southern Africa.^1^ The dominant histological subtype here is esophageal squamous cell carcinoma (ESCC). Its etiological factors in Africa remain understudied despite this high‐risk region being reported over six decades ago.^2^, ^3^ In recent years, several research studies have been initiated to address this research gap, largely within the African EC consortium AfrECC.^4^ Among these studies are the ESCC African PrEvention research (ESCCAPE) case‐control studies in Kenya, Tanzania and Malawi. To date ESCCAPE results have highlighted the role of alcohol, poor oral health and hot beverage consumption,^5^, ^6^, ^7^ while other African studies have identified dietary factors (such as daily spicy chilies and salted foods) and indoor air pollution.^8^

**FIGURE 1:**
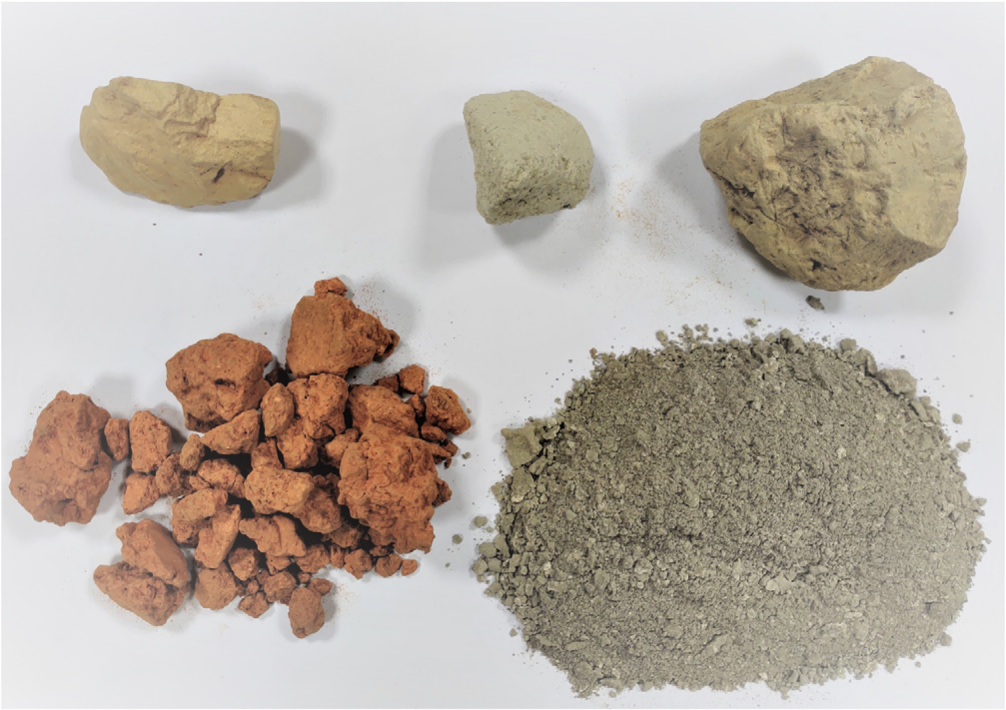
Photographs of geophagia tablets or samples from marketplaces in East and Southern Africa [Color figure can be viewed at wileyonlinelibrary.com]

With a multicausal etiology, a hypothesized contributor to ESCC risk in East Africa is geophagia. Geophagy (also known as pica) is the intentional practice of eating earth or soil and is a habit that spans all continents of the world.^9^ Universally, geophagia is known to be practiced mostly by young adults and pregnant women^10^ and, as a result, most geophagia studies have focused on pregnant women.^10^, ^11^, ^12^, ^13^, ^14^, ^15^ The prevalence of geophagia consumption in Africa varies considerably within and across countries. In women, in 2009, Ogbonnaya reported a prevalence of 63% in Kenya,^16^ while in Tanzania Kawai reported a prevalence of 29%.^17^ In these countries the practice is known as *kula udongo* in kiSwahili, while in Malawi the practice of geophagia, *kudya dothi* in chichewa, is considered culturally as a confirmatory sign of pregnancy.^18^ In contrast to women, there are limited studies on geophagia involving men, presumably because the prevalence is low. For familiarity with this exposure, examples of the geophagia tablets that can be purchased in marketplaces and on street stalls in these East Africa countries are shown in Figure 1.

Studies of the health effects of geophagia have largely focused on this practice as a response to and as a risk factor for iron‐deficiency anaemia.^17^, ^19^ To our knowledge, there are no studies that have examined the association of geophagia with risk of ESCC. Biological mechanisms for an association, if present, may be through physical damage to the esophageal mucosa due to the coarseness of the soil (which also cause stomach pains)^20^ or through exposure to infectious, chemical or radioactive elements in soil, such as silica, Helminth infections or heavy metals.^21^ These pathways would imply that any effect of geophagia on ESCC risk would be dependent on the properties of the soil being consumed, and thus may be setting specific. Of possible relevance here are studies reporting increased risk of ESCC among cement, concrete or construction workers,^22^, ^23^ which are heavily exposed to dust. Thus, with geophagia being prevalent in young women (ie, of reproductive age: <50 years) in East Africa, we hypothesized that this practice might contribute to the ESCC burden particularly in this group. With this background, to the best of our knowledge this is the first study to investigate geophagia as a potential risk factor of ESCC in East Africa.

## 2 METHODS

### 2.1 Study design and ethical approval

Our study is part of the ESCCAPE multicountry case‐control study of ESCC in Kenya, Malawi and Tanzania. Cases were histologically or clinically confirmed patients newly diagnosed with ESCC at Moi Teaching and Referral Hospital in Eldoret, Kenya, Queen Elizabeth Central Hospital in Blantyre, Malawi and Kilimanjaro Christian Medical Centre (KCMC) in Moshi, Tanzania (Figure S1).

Recruitment of controls was conducted to ensure frequency matching on the age and sex distribution of cases. They were recruited from hospital visitors and out/inpatients from the same hospital as cases, excluding patients with a history of cancer or digestive diseases. All participants were age 18 years or older at the time of diagnosis/interview. Recruitment took place during the years 2015‐2018 in Kenya, 2015‐2020 in Tanzania and 2017‐2020 in Malawi. These three hospitals are each tertiary or referral institutions, but noteworthy are their locations outside of capital cities of their country, thus patients originate from more local areas than had these been capital city national referral centers.

### 2.2 Exposure data

In all three countries, participation in ESCCAPE involved completion of an interviewer‐administered questionnaire and providing biospecimen. The questionnaire answers were immediately captured on a tablet or smartphone. Kenya was the first ESCCAPE study and had its own questionnaire, while in Tanzania and Malawi, the study commenced later and in both of these countries we implemented the same questionnaire. Data on a wide range of factors on sociodemographic characteristics and known or putative ESCC risk factors were collected in both questionnaires. Central to this article, in Tanzania and Malawi, participants were asked “Have you ever eaten soil?,” where the response options were sex‐specific. In men they were (1) “No,” (2) “Yes, but rarely (less than 10 times),” (3) “Yes, I eat it regularly” and (4) “Prefer not to answer.” Among men who answered informatively (ie, excluding (4)), this exposure was analyzed as regular (3) versus no/rarely (1 + 2), as the latter two categories have similar cumulative levels of geophagia. In women, the options in response to the geophagia question were: (1) “No,” (2) “Yes, only when I was pregnant,” (3) “Yes, I eat it regularly” and (4) “Prefer not to answer.” In Tanzanian women, regular geophagia was rare (1 control, 7 cases), thus the exposure was analyzed as (pregnancy or regular [2 + 3] vs never use), while in Malawian woman all three categories were prevalent and were thus analyzed separately. In this latter group, “regularly” practicing geophagia is expected to reflect greater exposure than “pregnancy only” geophagia, however with a mean parity of 5.5, if women with many pregnancies practiced geophagia for many months per pregnancy, their total exposure may be high. We thus also split the “pregnancy only” geophagia category into pregnancy only geophagia among women who had up to four pregnancies, and pregnancy only geophagia among women who had more than four pregnancies. In Kenya, geophagia was only asked in the main study phase (not in the pilot phase) and it was asked only to women who had been pregnant. They were asked “Did you eat soil or clay when you were pregnant?” and responses were “No,” “Yes” and “Do not know.”

The questionnaire also included information on other risk factors for ESCC which may confound the ESCC‐geophagia association if they were also associated with geophagia use. They included tobacco, alcohol, hot beverage consumption and socio‐demographic characteristics (age, sex, education and occupation). Data on self‐reported HIV status were also collected.

### 2.3 Statistical analysis

We first examined the categorical distribution of the primary exposure of interest, geophagia. We presented these distributions stratified by case/control status, country and sex (male and female, female only in Kenya). Secondly, to understand the population prevalence and patterns of geophagia practice and identify factors that might lead to confounding of any geophagia‐ESCC association, we examined how geophagia habits in controls varied by socio‐economic factors and ESCC risk factors (alcohol drinking and smoking habits), tested using chi‐squared statistics. Thirdly, we assessed the association of geophagia with ESCC using logistic regression models to estimate odds ratios (ORs) and their 95% confidence intervals (95% CI). The minimally adjusted model (mOR) included adjustment for the matching factors age and sex, to account for small imbalances in frequency matching. In the adjusted model (aOR), we additionally controlled for known ESCC risk factors/markers: highest level of education (none/partial primary, completed primary, secondary school and above), main occupation (farming vs nonfarming), marital status (unmarried vs married), parity (0‐4, 5‐7 and 7+ children), HIV status (positive, negative and unknown), place of residence (urban vs rural), smoking (yes vs no) and alcohol consumption (yes vs no). For all the above models, analyses were first conducted separately for country‐sex strata. We also pooled data for women into a single model, adjusted for country and including an interaction term between age group and country. Sensitivity analyses were conducted by type of control (hospital patient or visitor) to investigate the impact of the choice of the control group, and we also examined the potential effect modification by age (<50 vs 50+ years), because the younger (<50 years) age group is closer to the time of geophagia exposure and the young onset of ESCC in East Africa is a feature not found in other parts of the world.

A final analysis examined whether geophagia habits were associated with the anatomical location (upper, middle and lower) of the tumor within the esophagus, as recorded at endoscopy (chi‐squared test). All the data cleaning and statistical analysis were performed in Stata v14.0 (Stata Corp, College Station, Texas).

## 3 RESULTS

### 3.1 Participant characteristics

Table 1 provides sociodemographic characteristics of the study participants according to country and case‐control status. In total, 1947 participants were asked about geophagia: 1003 controls and 944 cases, of which females made up 396 (42%) of cases and 431 (43%) of controls. The mean age of cases ranged from 57 years (SD = 14.3) in Malawi/Kenya to 64 years (SD = 14.0) in Tanzania. Most controls 477 (48%) and cases 461 (59%) had either no formal or partial primary education. Most participants were rural dwellers and farming was a common occupation. In each of the three countries, tobacco smoking and alcohol use were both more common in cases than controls (absolute differences of 20%‐40% points). More than one‐half of controls were visitors to the study facilities (n = 578, 58%). In Malawi and Kenya, all cases were recruited at endoscopy, and in Tanzania the majority (70%) were endoscopy‐confirmed while the remaining were confirmed via barium swallow or clinically with the specific symptoms of severe dysphagia.

**TABLE 1:** Socio‐demographic characteristics of study participants who were asked about geophagia: ESCCAPE case–control studies (1003 cases and 944 controls)

	Country					
	Tanzania		Malawi		Kenya (women only^a^)	
Variable n (Column %)	Control (n = 313)	Case (n = 310)	Control (n = 593)	Case (n = 539)	Control (n = 97)	Case (n = 95)
Sex						
Men	237 (76)	237 (76)	335 (56)	311 (58)	0 (0)	0 (0)
Women	76 (24)	73 (24)	258 (44)	228 (42)	97 (100)	95 (100)
Age, years						
Mean (SD)	62.4 (14.2)	63.7 (14.1)	55.5 (14.7)	56.8 (14.3)	56.8 (15.1)	58.9 (14.4)
Type of control						
Non‐hospital	239 (76)	–	308 (52)	–	32 (33)	–
Hospital in/out‐patient	74 (24)	–	285 (48)	–	64 (67)	–
Smoking^b^						
No	259 (83)	129 (42)	452 (76)	316 (57)	88 (91)	69 (73)
Yes	54 (17)	181 (58)	140 (24)	223 (41)	9 (9)	26 (27)
Alcohol						
No	171 (55)	75 (24)	341 (58)	281 (51)	79 (81)	48 (51)
Yes	142 (45)	235 (76)	252 (43)	258 (48)	18 (19)	47 (50)
Religion						
Christian	201 (86)	187 (88)	526 (89)	445 (83)	96 (99)	91 (96)
Muslim	33 (14)	24 (11)	50 (8)	59 (11)	0 (0)	0 (0)
Other^c^	1 (0.4)	2 (0.9)	17 (3)	34 (6)	1 (1)	4 (4)
Education						
None/partial primary	90 (29)	152 (40)	325 (55)	346 (64)	62 (64)	63 (67)
Completed primary	162 (52)	118 (38)	82 (14)	67 (12)	15 (16)	17 (18)
Secondary and above	61 (20)	40 (13)	186 (31)	126 (23)	20 (21)	15 (16)
Occupation						
Farming	250 (80)	266 (86)	208 (35)	229 (43)	59 (61)	63 (66)
Non farming	63 (20)	44 (14)	385 (65)	310 (58)	38 (39)	32 (34)
Parity						
0–4	129 (41)	107 (35)	248 (42)	239 (44)	29 (30)	32 (34)
5–7	139 (44)	141 (45)	244 (41)	210 (39)	45 (46)	45 (47)
7+	45 (15)	62 (20)	101 (17)	90 (17)	23 (24)	18 (19)
Mean (SD)	5.6 (3.1)	6.0 (3.5)	5.5 (3.1)	5.4 (3.3)	5.9 (3.1)	5.9 (2.9)
HIV status						
Positive	6 (2)	14 (5)	124 (21)	131 (24)	10 (10)	11 (12)
Negative	222 (71)	162 (52)	396 (67)	364 (68)	72 (74)	73 (77)
Unknown	85 (27)	134 (43)	73 (12)	44 (8)	15 (16)	11 (12)
Residence						
Urban	45 (14)	33 (11)	253 (43)	207 (38)	80 (83)	90 (95)
Rural	268 (86)	277 (89)	340 (57)	332 (62)	17 (18)	5 (5)
Ethnicity^d^						
Country‐specific groups	Chagga 227 (73)	199 (64)	Lomwe 171 (29)	146 (27)	Kalenjin 58 (60)	52 (55)
	–	–	Ngoni 99 (17)	95 (18)	Luhya 12 (12)	23 (24)
	–	–	Yao 86 (15)	87 (16)	–	–
	–	–	Chewa 60 (10)	66 (12)	–	–
Other	86 (28)	111 (36)	177 (30)	145 (27)	27 (28)	20 (21)

^a^ Only women were asked about geophagia in Kenya.

^b^ No response in Malawi.

^c^ Other: None/African traditional/refuse.

^d^ Other ethnicities (Tanzania: Non‐Chagga; Malawi: Sena, Manganja, and others; Kenya: Luo, Kikuyu, Kisii and others).

### 3.2 Geophagia prevalence

Among all 1947 participants asked about their past geophagia habits, only 18 (0.9%) responded that that they preferred not to answer this question—they are excluded hereafter. Geophagia was far more common in women than men, thus the main Table 2 presents geophagia prevalence by case/control status in women and, among female controls, correlates of the geophagia practice. The same data on geophagia prevalence in male cases and male controls are contained in Table S1. In all three countries, geophagia prevalence among women was reported by at least one‐third of both cases and controls. However, there were few factors which were correlated with geophagia prevalence. Notably, geophagia was practiced by women regardless of urban/rural residency, parity, religion or marital status and did not vary by these factors or by alcohol use or tobacco smoking. Associations were seen with age in Malawian participants, where a regular geophagia habit was twice as likely under age 40 than above, in both sexes (*P*‐trend = .03). Geophagia prevalence differed substantially by the type of control participant. In both men and women, regular geophagia was twice as common among hospital patient controls than among hospital visitor controls. In Kenyan and Tanzanian female controls, a similar trend was seen with an absolute 20%+ more hospital patients than hospital visitor reporting pregnancy/regular geophagia. In Tanzanian men, geophagia was reported by two cases and no controls, thus the exposure was too rare to be analyzed further. Regular geophagia was reported by 10% of Malawian men (controls).

**TABLE 2:** Country‐specific prevalence of geophagia in female cases and controls, and among controls, by sociodemographic factors

	Country										
	n (Row %)	Kenya			Tanzania			Malawi			
Variable	Category	Never	Preg‐nancy	Total	Never	Preg‐nancy only/regular	Total	Never (%)	Pregnancy only (%)	Regular (%)	Total
Status	Cases	61 (64)	34 (36)	95	41 (61)	27 (37)	68	125 (55)	65 (29)	37 (16)	227
	Controls	55 (57)	42 (43)	97	52 (71)	21 (28)	73	132 (51)	96 (37)	30 (12)	258
	*P‐value*			*.29*			*.14*				*.09*
*Among controls*											
Type of control	Hospital visitors	22 (69)	10 (31)	32	44 (80)	11 (20)	55	72 (50)	60 (42)	12 (8)	144
	Hospital patients	32 (50)	32 (50)	64	8 (44)	10 (56)	18	60 (53)	36 (32)	18 (16)	114
	*P‐value*			*.08*			*<.01*				*.58*
Age	18 to <40	6 (67)	3 (33)	9	5 (83)	1 (17)	6	12 (35)	15 (44)	7 (21)	34
	40‐50	8 (44)	10 (56)	18	9 (75)	3 (25)	12	34 (53)	23 (36)	7 (11)	64
	50+	41 (59)	29 (41)	70	38 (69)	17 (31)	55	86 (54)	58 (36)	16 (10)	160
	*P‐value*			*.89*			*.44*				*<.01*
Parity	0–4	17 (59)	12 (41)	29	30 (79)	8 (21)	38	53 (49)	36 (33)	19 (18)	108
	5–7	21 (47)	24 (53)	45	16 (62)	10 (38)	26	59 (57)	38 (37)	6 (6)	103
	7+	17 (74)	6 (26)	23	6 (67)	3 (33)	9	20 (43)	22 (47)	5 (11)	47
	*P‐value*			*.34*			*.22*				*.53*
Education	None/partial primary	36 (58)	26 (42)	62	16 (59)	11 (41)	27	89 (53)	59 (35)	19 (12)	167
	Completed primary	8 (53)	7 (47)	15	28 (78)	8 (22)	36	16 (52)	10 (32)	5 (16)	31
	≥Secondary	11 (55)	9 (45)	20	8 (80)	2 (20)	10	27 (45)	27 (45)	6 (10)	60
	*P‐value*			*.75*			*.09*				*.46*
Religion	Christian	55 (57)	41 (43)	96	28 (73)	10 (26)	38	118 (51)	88 (38)	27 (11)	233
	Muslim	–	–	–	7 (88)	1 (13)	8	10 (56)	6 (33)	2 (11)	18
	Other	0	1 (100)	1	–	–	–	4 (57)	2 (29)	1 (14)	7
	*P‐value*			*.26*			*.42*				*.76*
Marital	Married	36 (53)	32 (47)	68	46 (73)	17 (27)	63	68 (50)	56 (41)	13 (9)	137
Status	Unmarried	19 (66)	10 (34)	29	6 (60)	4 (40)	10	64 (53)	40 (33)	17 (14)	121
	*P‐value*			*.26*			*.41*				*.88*
HIV	Positive	5 (50)	5 (10)	10	1 (100)	0	1	22 (38)	25 (43)	11 (19)	58
	Negative	41 (57)	31 (43)	72	42 (82)	9 (18)	51	90 (54)	62 (37)	15 (9)	167
	Unknown	9 (60)	6 (40)	15	9 (43)	12 (57)	21	20 (61)	9 (27)	4 (12)	33
	*P‐value*			*.68*			*.65*				*.10*
Residence	Urban	46 (58)	34 (43)	80	10 (67)	5 (33)	15	54 (48)	47 (42)	11 (10)	112
	Rural	9 (53)	8 (47)	17	42 (72)	16 (28)	58	78 (53)	49 (34)	19 (13)	146
	*P‐value*			*.73*			*.67*				*.82*
Smoking	No	51 (58)	37 (42)	88	52 (71)	21 (29)	73	125 (52)	90 (37)	26 (11)	241
	Yes	4 (44)	5 (56)	9	–	–	–	6 (38)	6 (38)	4 (24)	16
	*P‐value*			*.44*			*–*				*.89*
Alcohol	No	44 (56)	35 (44)	79	35 (74)	12 (26)	47	117 (52)	82 (37)	24 (11)	223
	Yes	11 (61)	7 (39)	18	17 (65)	9 (35)	26	15 (43)	14 (40)	6 (17)	35
	*P‐value*			*.68*			*.42*				*.20*

*Note*: *Chi‐square *P*‐value. *P* is *P*‐value for difference between in geophagia prevalence between cases and controls, or, in controls, *P*‐value for difference by HIV status (restricted to known status), urban/rural, smoking, alcohol and *P*‐value for trend in age, parity and education.

### 3.3 Geophagia factors associated with esophageal squamous cell carcinoma

Associations between geophagia and ESCC risk are shown in Table 3, by country and sex. Among men, only Malawi could be analyzed, in whom there was no evidence that regular geophagia was associated with ESCC risk (aOR: 0.95, 95% CI: 0.53, 1.69). Among women, pregnancy‐only compared to never geophagia was not consistently associated with ESCC risk across the three countries, with decreased ORs in Kenya and Malawi and an increased OR in Tanzania (for pregnancy‐only and regular use combined). Regular geophagia use in Tanzania was not analyzed separately, as only reported by 5 cases and 1 control (not shown in table). In Malawi, the aOR for regular versus never geophagia was 1.13, with a wide 95% CI of 0.64 to 2.00. As geophagia was only common in females, an all‐female model was constructed which showed 25% increased risk for regular versus never geophagia (aOR: 1.25, 95%: CI: 0.70, 2.22) for the three countries combined. If the reference group is taken as women with a pregnancy‐only geophagia habit, then a regular habit was associated with an aOR of 1.60 (95% CI: 0.86, 2.96; not shown in table).

**TABLE 3:** Country and sex‐specific odds ratios for ESCC associated with the practice of geophagia

Sex	Country	Age‐sex adjusted	No. cases/controls	Age‐adjusted OR (95% CI)	Fully‐adjusted^a^ OR (95% CI)
Men	Malawi	Never/rare	280/298	1	1
		Regularly	28/32	0.98 (0.57, 1.68)	0.95 (0.53, 1.69)
	Tanzania	*Geophagia was rare in men*		–	–
	Kenya	*Geophagia not asked in men*			
Women	Tanzania	Never	41/52	1	1
		Pregnancy only + Regularly^b^	27/21	1.61 (0.79, 3.29)	1.66 (0.77, 3.55)
	Kenya	Never	61/55	1	1
		Pregnancy only	34/42	0.74 (0.41, 1.33)	0.71 (0.38, 1.34)
					
	Malawi	Never	125/132	1	1
		Pregnancy only	65/96	0.73 (0.48, 1.09)	0.73 (0.48, 1.11)
		Regularly	37/30	1.32 (0.76, 2.27)	1.13 (0.64, 2.00)
Women	All^c^	Never	227/239	1	1
		Pregnancy only	121/158	0.83 (0.61, 1.12)	0.88 (0.64, 1.22)
		Pregnancy only and parity ≤4	43/56	0.88 (0.56, 1.38)	0.93 (0.57, 1.48)
		Pregnancy only and parity 4+	78/102	0.80 (0.57, 1.13)	0.82 (0.54, 1.25)
		Regularly	42/31	1.48 (0.89, 2.44)	1.25 (0.70, 2.22)
Women	Malawi	Never	125/132	1	
		Pregnancy only	65/96	0.73 (0.48, 1.09)	0.73 (0.48, 1.11)
		Pregnancy only and parity ≤4	27/36	0.83 (0.47, 1.49)	0.89 (0.49, 1.61)
		Pregnancy only and parity 4+	38/60	0.67 (0.42, 1.08)	0.65 (0.39, 1.06)
		Regularly	37/30	1.32 (0.77, 2.29)	1.14 (0.65, 2.01)
*Subset and sensitivity analysis in women only (all countries combined)*					
Control type	All cases vs hospital patient controls	Never	227/99	1	1
		Pregnancy only	121/77	0.67 (0.46, 0.98)	0.75 (0.50, 1.12)
		Regularly	42/19	0.94 (0.52, 1.71)	0.87 (0.46, 1.65)
Control type	All cases vs hospital visitor controls	Never	227/138	1	1
		Pregnancy only	121/81	0.97 (0.68, 1.40)	1.06 (0.71, 1.58)
		Regularly	42/12	2.41 (1.22, 4.76)	1.68 (0.80, 3.54)
Age	<50 years	Never	59/74	1	1
		Pregnancy only	50/55	1.15 (0.69, 1.93)	1.20 (0.68, 2.14)
		Regularly	16/14	1.56 (0.70, 3.56)	1.25 (0.55, 2.86)
Age	50+ years	Never	168/164	1	1
		Pregnancy only	71/103	0.69 (0.47, 1.00)	0.71 (0.47, 1.08)
		Regularly	26/17	1.50 (0.78, 2.91)	1.09 (0.52, 2.25)

*Note*: For the OR, regularly was combined with pregnancy only, due to too few participants.

^a^ Fully adjusted models included age (design factor), smoking habit, alcohol drinking, marital status and religious belief with adjustment for country in the “all women” models.

^b^ Small numbers of regular users, hence, this overall result is driven by pregnancy‐only users.

^c^ Adjusted for country.

In a sensitivity analysis of the geophagia association with ESCC by type of control, associations based on a comparison of cases with hospital patients showed slightly decreased ORs, but with wide confidence intervals (aOR = 0.75 in pregnancy only vs never use and aOR = 0.87 for regular vs never use). However, when comparing cases to hospital visitor controls, a regular versus never geophagia habit was associated with an increased risk of ESCC (aOR: 1.68 [95% CI: 0.80, 3.54]). Effect modification by age was not significant, though the OR for regular geophagia among women under age 50 (aOR: 1.25, 95% CI: 0.55, 2.86) was much stronger than at ages 50 and over (aOR: 1.00, 95% CI: 0.52, 2.25).

### 3.4 Tumor location among cases

Among the Malawian men, the location of esophageal tumor was in the lower thoracic (30‐40 cm)/gastro‐esophageal junction for more than 50% of cases, 29% in the mid esophagus and 21% in the upper (Table 4). This distribution did not differ by geophagia use (*P* = .66). Among women, more tumors were located in the upper esophagus than among men, but there was also no association between tumor location and geophagia (*P* = .39). Similarly there no associations in Kenyan or Tanzanian women.

**TABLE 4:** Distribution of the tumor location within the esophagus, among esophageal cancer cases in Malawi, by past geophagia habits (never, rarely or regular)

	Tumor location *N* (row %)^a^			Total	Chi‐squared test for differences (P‐value)
Case group	Upper	Middle	Lower		
*Malawi men*					
Never/rare	43 (15)	75 (27)	160 (58)	249 (100)	0.66
Regular	6 (21)	8 (29)	14 (50)	28 (100)	
*Malawi women*					
Never	29 (23)	43 (34)	53 (43)	125 (100)	
Pregnancy only	13 (20)	28 (43)	24 (37)	65 (100)	0.39
Regular	8 (22)	8 (22)	21 (56)	37 (100)	
*Kenyan women* ^b^					
Never	25 (44)	18 (32)	14 (25)	57 (100)	0.42
Pregnancy only	10 (30)	14 (42)	9 (27)	33 (100)	
*Tanzanian women* ^b^					
Never	8 (24)	17 (50)	9 (26)	34 (100)	0.56
Pregnancy only or regular	3 (15)	13 (65)	4 (20)	20 (100)	

^a^ Analyses could not be conducted of Tanzanian male cases due to too few who had practiced geophagia (7 rare users and 2 regular).

^b^ Fourteen missing tumor location in Tanzania and five in Kenya.

## 4 DISCUSSION

### 4.1 Main findings

The present study is the first examination of the association of geophagia (soil eating) habits with risk of ESCC in East Africa, a setting where geophagia is commonly practiced among women and where ESCC incidence is high. The study revealed that a regular geophagia habit was too rare in men to possibly contribute to the ESCC burden. We confirmed the higher prevalence of this habit in women, as expected, but we did not find consistent evidence that pregnancy‐only or regular geophagia increased the ESCC risk. Our study suggests that the physical effect of geophagia is not a major contributor to ESCC burden in East Africa. A caveat to this overall interpretation is that the study had limited statistical power to examine associations separately at younger ages (<50 years), where there was a weak suggestion of a stronger effect.

### 4.2 Comparison with other studies

While no other previous study has reported on ESCC risk related to geophagia, we can make comparisons with other studies of ever‐geophagia prevalence among our controls. Our estimates of 29%, 43% and 49% ever‐geophagia in Tanzanian, Kenyan and Malawian female controls are slightly lower than previous studies in Tanzania and Kenya reporting between 64% and 74%,^24^, ^25^ but regional and cultural variations within countries may be real. The lack of a socioeconomic or urban/rural gradient in geophagia prevalence in the present Tanzania and Malawian settings has also reassuringly been reported by social and anthropological studies of this habit especially among pregnant women,^17^, ^19^ while higher geophagia prevalence in less educated women in western Kenya has also been reported by others.^26^ However in our study, we observed much lower geophagia prevalence among hospital visitor than among hospital patient controls, and consequently the comparison of cases to hospital visitor controls suggested a positive geophagia‐ESCC association. This difference in geophagia prevalence by control type may be real, if hospital visitors are from population groups who truly had lower geophagia prevalence, but with few strong sociocultural variations in the habit, this seems an unlikely explanation. The lower prevalence may instead be due to differential under‐reporting of this habit among this group of hospital visitor controls. Some women may be reluctant to admit the habit, since some antenatal care clinics now discourage it.^27^ Possibly related to and supporting a general issue of under‐reporting, we also observed slightly lower geophagia prevalence among cases in Kenya who were interviewed by the male interviewer than in those interviewed by the female interviewer (data not shown). However in Malawi, all interviewers were women. Under‐reporting of geophagia has also previously been reported in studies related to pregnancies, citing embarrassment, lack of cultural sensitivities and different perceptions and norms. Given the uncertainties in defining an optimal control group, investigations on geophagia from other on‐going studies in the region will be useful for other groups to report upon, and their investigation of these possible influences on the findings.

### 4.3 Study strengths and limitations

Geophagia exposure assessment was limited in detail as the ESCCAPE questionnaire contained just one geophagia question on soil‐eating practices of the participants. As a result, we could not account for type of geophagia, its chemical composition including of toxic elements, texture, quantity consumed (number of tablespoons per day) and durations of use. Our exposure assessment was thus crude in nature with respect to the dose/intensity data, which alongside information on the soil coarseness (varieties are seen in Figure 1), would be relevant metrics to investigate a physical irritation pathway of action. In contrast, to investigate whether specific chemical constituents of geophagy mediated ESCC risk, information on the type of geophagy and where it was purchased should have been noted, ideally with the collection and analysis of actual samples. Notably, Msoffe et al. have analyzed the distribution of geophagy between various original sources and marketplaces across Tanzania, as well as the chemical contents of geophagia samples from these markets. Their findings demonstrate that the chemical composition of geophagia is highly heterogeneous, with some but not all containing toxic elements including arsenic, cadmium, lead and mercury.^28^ Thus “geophagia” is a very heterogeneous exposure entity. With a crude exposure assessment in the present study, we can at most be examining any effects due to physical irritation while the chemical exposures included across widespread areas of the three countries are likely to vary substantially. Additionally, there was limited statistical power to detect small ORs in each sex‐country stratum (see Table S2). Other inherent limitations of the case‐control study design include the possibility of control selection bias and misclassification of the exposure status, as already mentioned, in particular the under‐reporting of geophagia. Strengths of the study are a large ESCC study which has already led to the identification of several risk factors.^5^, ^6^, ^7^ Further, the potential selection bias when recruiting from a capital city tertiary hospital—of hospital controls residing nearby while cases originate from afar—was minimized.

### 4.4 Implications

Using this crude assessment of geophagia, it does not appear to be a major contributor to the high incidence ESCC belt in Eastern Africa—certainly not in men and unlikely in women. A possibility of a positive association remains among females at young ages (<50 years), or from geophagia from certain locations or with certain contents. Thus future research would be prudent to continue to collect basic exposure data on this factor. Further to recent acceptability studies of the cytosponge in Tanzania, this pill‐on‐a string for esophageal cell collection may also offer a unique tool to examine the impact of geophagia on the esophageal epithelium, accompanied by analytical studies of the chemical constituents of the particular soil being consumed.

## CONFLICT OF INTEREST

The authors declare no conflicts of interest.

## ETHICS STATEMENT

The study was approved by the Institutional Research and Ethics Committee of Moi University in Kenya, National Health Sciences Research Committee in Malawi, Kilimanjaro Christian Medical University College Research Ethics Committee and National Health Research Ethics Review Committee in Tanzania, and by the International Agency for Research on Cancer (IARC). All participants provided written or thumbprint informed consent; consent forms and participant information sheets were provided in national languages (swahili or chichewa).

## Supporting information

**Appendix S1**: Supporting Information

## Data Availability

Data for our study is available in collaboration with the ESCCAPE study through https://esccape.iarc.fr/.
