# Supplementary material for: Geophagia and risk of squamous cell esophageal cancer in the African esophageal cancer corridor: Findings from the ESCCAPE multicountry case‐control studies
Source: Int J Cancer. 2021 May 28;149(6):1274–83. doi: 10.1002/ijc.33688 (PMC8411422; doi:10.1002/ijc.33688)

Supplementary Materials

Geophagia and risk of squamous cell esophageal cancer in the African esophageal cancer corridor - Findings from the ESCCAPE multi-country case-control studies

Clement T Narh[1,2], Charles P Dzamalala[3], Blandina T Mmbaga[4,5], Diana Menya[6], Yohannie Mlombe[3], Peter Finch[3], Gissela Nyakunga[5], Joachim Schüz[1] and Valerie McCormack[1], on behalf of the ESCCAPE team*

* The ESCCAPE team is listed in the Acknowledgements section.

Table of contents

Supplementary Table 1: Country and prevalence of geophagia in male controls, overall and by socio-demographic characteristics

Supplementary Table 2: Odds ratios detectable with the number of cases and geophagia prevalence in ESCCAPE, by sex and country

Supplementary Figure 1: ESCCAPE esophageal cancer case-control study recruitment centres in Kenya, Tanzania and Malawi, Eastern Africa

Supplementary tables

Supplementary Table 1: Country and prevalence of geophagia in male controls, overall and by socio-demographic characteristics

		Country					
	n (Row %)	Tanzania			Malawi		
**Variable**	**Category**	**Never/ Irregular**	**Regular**	**Total**	**Never/ Irregular**	**Regular**	**Total**
Status	Cases	234 (99)	2 (<1)	236	280 (91)	28 (9)	308
	Controls	237 (100)	0 (0)	237	298 (90)	32 (10)	330
	P-value			*0.16*			*0.79*
**Among controls**							
Type of Control	Hospital visitors	-	-	-	152 (93)	11 (7)	163
	Hospital patients	-	-	-	146 (87)	21 (13)	167
	P-value	-	-	-			*0.07*
Age	18 to <40	-	-	-	37 (80)	9 (20)	46
	40-50	-	-	-	70 (92)	6 (8)	76
	50+	-	-	-	191 (92)	17 (8)	76
	P-value	-	-	-			*0.03*
Parity	0-4	-	-	-	115 (85)	21 (15)	136
	5-7	-	-	-	134 (95)	7 (5)	141
	7+	-	-	-	49 (92)	4 (8)	53
	P-value	-	-	-			*0.02*
Education	None/partial primary	-	-	-	143 (92)	13 (8)	156
	Completed primary	-	-	-	47 (94)	3 (6)	50
	≥ Secondary	-	-	-	108 (87)	16 (13)	124
	P-value	-	-	-			*0.60*
Religion	Christian	-	-	-	260 (90)	29 (10)	289
	Muslim	-	-	-	30 (94)	2 (6)	32
	Other	-	-	-	8 (89)	1 (11)	9
	P-value	-	-	-			*0.71*
Marital Status	Married	-	-	-	249 (91)	26 (9)	275
	Unmarried	-	-	-	49 (89)	6 (11)	55
	P-value	-	-	-			*0.74*
HIV	Positive	-	-	-	58 (89)	7 (11)	65
	Negative	-	-	-	206 (91)	20 (9)	226
	Unknown	-	-	-	34 (87)	5 (13)	39
	P-value	-	-	-			*0.65*
Residence	Urban	-	-	-	123 (89)	15 (11)	138
	Rural	-	-	-	175 (91)	17 (9)	192
	P-value	-	-	-			*0.54*
Smoking	No	-	-	-	189 (91)	18 (9)	207
	Yes	-	-	-	109 (89)	14 (11)	123
	P-value	-	-	-			*0.43*
Alcohol	No	-	-	-	111 (94)	7 (6)	118
	Yes	-	-	-	187 (88)	25 (12)	212
	P-value	-	-	-			*0.89*

*chi-square p-value. Footnote: P is P-value for difference between in ever-geophagia prevalence between cases and controls, or, in controls, P-value for difference by HIV status (restricted to known status), urban/rural, smoking, alcohol and P-value for trend in age, parity and education

Supplementary Table 2: Odds ratios detectable with the number of cases and geophagia prevalence in ESCCAPE, by sex and country

Country	Sex	ESCCAPE Sample size[a] Number of cases	Prevalence (%)[b]	OR detectable
Tanzania	Male	237	0.8	5.3
	Female	73	28.8	2.6
Malawi	Male	328	20.4	1.6
	Female	256	49.2	1.6
Kenya	Male	-	-	-
	Female	97	43.3	2.3
Overall	Male	565	12.2	1.6
	Female	426	44.4	1.5

a Number of controls and 1:1 case: control ratio
b Prevalence of geophagia in controls

Supplementary figure

Supplementary Figure 1: ESCCAPE esophageal cancer case-control study recruitment centres in Kenya, Tanzania and Malawi, Eastern Africa.

© OpenStreetMap and contributors, CC-BY-SA
(http://www.openstreetmap.org/, and http://creativecommons.org/licenses/by-sa/2.0/)

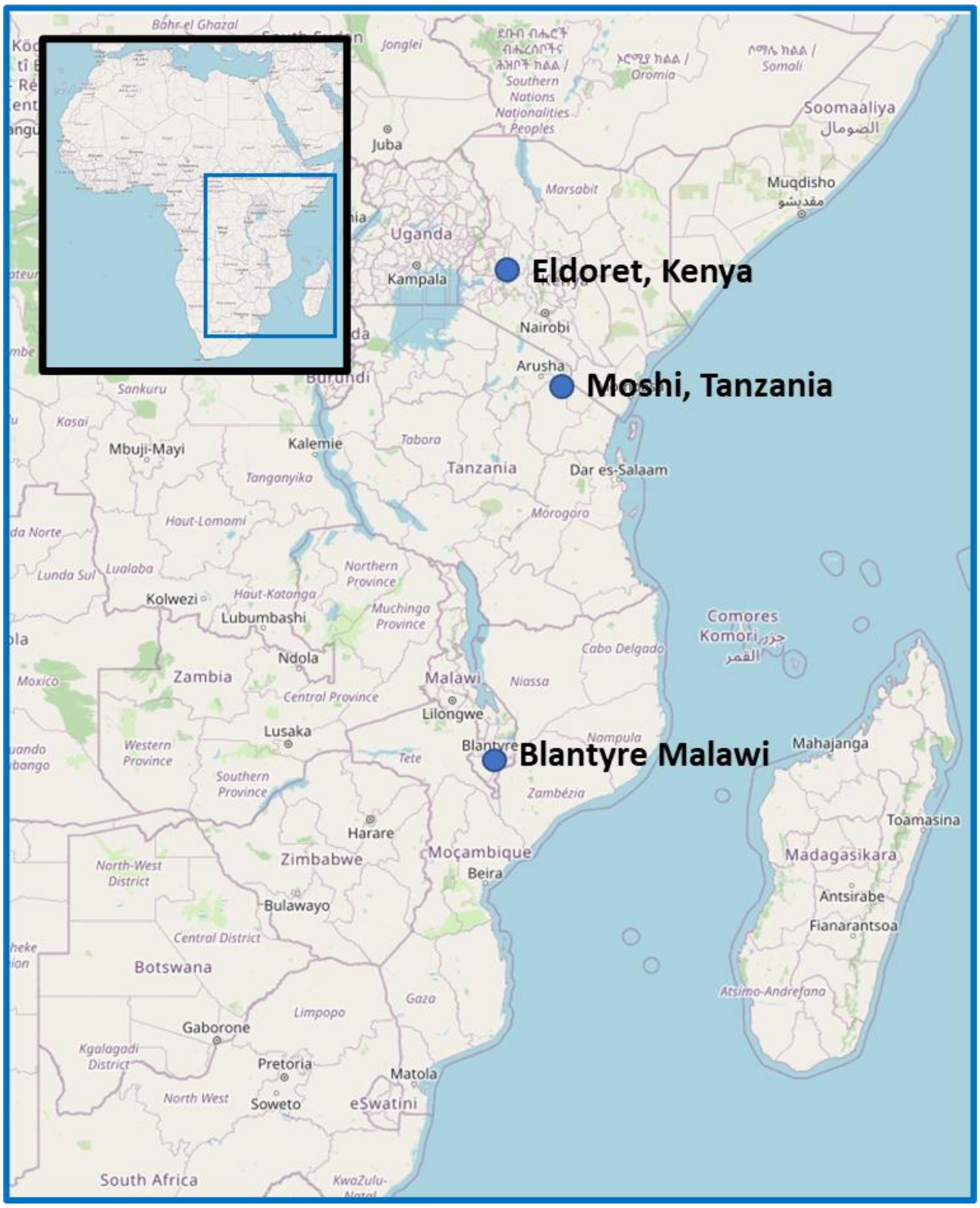

Supplement: Supplementary file 1 — Appendix S1: Supporting Information [file IJC-149-1274-s001.pdf]
